# Striatonigrostriatal circuit architecture for disinhibition of dopamine signaling

**DOI:** 10.1016/j.celrep.2022.111228

**Published:** 2022-08-16

**Authors:** Priscilla Ambrosi, Talia N. Lerner

**Affiliations:** 1 Department of Neuroscience, Northwestern University Feinberg School of Medicine, Chicago, IL 60611, USA; 2 Northwestern University Interdepartmental Neuroscience Program (NUIN), Evanston, IL 60208, USA; 3 Lead contact

## Abstract

The basal ganglia operate largely in closed parallel loops, including an associative circuit for goal-directed behavior originating from the dorsomedial striatum (DMS) and a somatosensory circuit important for habit formation originating from the dorsolateral striatum (DLS). An exception to this parallel circuit organization has been proposed to explain how information might be transferred between striatal subregions, for example, from the DMS to the DLS during habit formation. The “ascending spiral hypothesis” proposes that the DMS disinhibits dopamine signaling in the DLS through a tri-synaptic, open-loop striatonigrostriatal circuit. Here, we use transsynaptic and intersectional genetic tools to investigate both closed- and open-loop striatonigrostriatal circuits. We find strong evidence for closed loops, which would allow striatal subregions to regulate their own dopamine release. We also find evidence for functional synapses in open loops. However, these synapses are unable to modulate tonic dopamine neuron firing, questioning the prominence of their role in mediating crosstalk between striatal subregions.

## In brief

Ambrosi and Lerner investigate a basal ganglia circuit—the ascending spiral—long hypothesized to underlie habit formation by allowing disinhibition of dopamine signaling in the dorsolateral striatum (DLS). They find that synaptic connectivity in the ascending spiral is present, but the circuit cannot support straightforward disinhibition of DLS dopamine signaling.

## Graphical Abstract

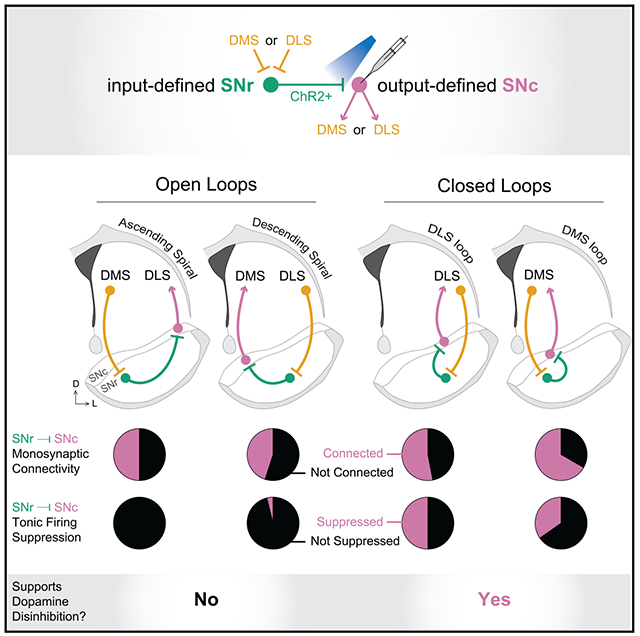

## INTRODUCTION

The striatum is well known for its roles in motor control and reinforcement learning. The dorsomedial striatum (DMS) is thought to be involved in goal-directed learning, while the dorsolateral striatum (DLS) is thought to be involved in motor skill acquisition and habit formation (Lipton et al., 2019; Yin and Knowlton, 2006). As animals are overtrained in a motor skill task (e.g., accelerating rotarod) or in an instrumental task designed to elicit habit (e.g., random interval training), their behavior becomes more stereotyped and less flexible, and dependence of the behavior shifts from the DMS to the DLS (Corbit et al., 2012; Derusso et al., 2010; Gremel and Costa, 2013; Sommer et al., 2014; Thorn et al., 2010; Yin et al., 2004, 2005a, 2005b, 2006, 2009).

Both the DMS and the DLS are richly innervated by dopamine (DA) neurons from the substantia nigra pars compacta (SNc). Although DA axonal fields in striatum are broad (Matsuda et al., 2009), there is topography within the nigrostriatal system that can allow for separate control of DA release in the DMS and the DLS (Farassat et al., 2019; Ikemoto, 2007; Joel and Weiner, 2000; Lerner et al., 2015). Indeed, DA neurons projecting to the DMS and those projecting to the DLS display distinct *in vivo* activity patterns (Brown et al., 2011; Hamid et al., 2021; Lerner et al., 2015; Seiler et al., 2022; Tsutsui-Kimura et al., 2020).

How distinct activity in DMS- and DLS-projecting DA neurons arises is a key question. One possibility is that these cells receive distinct inputs (Lerner et al., 2015). In particular, it has been widely hypothesized that DMS-DLS transitions observed during habit formation are regulated by an input circuit to DLS-projecting DA neurons termed the “ascending spiral” (Haber et al., 2000; Lerner, 2020; Lüscher et al., 2020; Yin and Knowlton, 2006). The premise of the ascending spiral hypothesis is that the DMS and the DLS are connected by a tri-synaptic circuit involving GABAergic neurons in substantia nigra pars reticulata (SNr) and DA neurons in the SNc. More specifically, DA neurons are thought to be under tonic inhibition from GABAergic neurons in the SNr; spiny projection neurons (SPNs) from the DMS can inhibit these SNr GABA cells and thus disinhibit DLS-projecting DA neurons, allowing for DA release in the DLS. The individual steps in this polysynaptic circuit (DMS→SNr, SNr→SNc, and SNc→DLS) are well established (Chevalier et al., 1985; Freeze et al., 2013; Tepper and Lee, 2007; Tepper et al., 1995). However, it is not necessarily the case that these individual connections link into a continuous polysynaptic circuit (DMS→SNr→SNc→DLS). Indeed, anatomical and electrophysiological work in other basal ganglia circuits supports a largely parallel organization of DMS and DLS subcircuits (Alexander et al., 1986; Lee et al., 2020; Mandelbaum et al., 2019). The idea that an ascending spiral through the midbrain DA system could be a major route of crosstalk between otherwise parallel circuits has been appealing to behavioral neuroscientists, but evidence of a functional circuit at the synaptic level is lacking.

Evidence for the ascending spiral circuit stems primarily from anatomical work done in non-human primates (Haber et al., 2000). Following the injection of retrograde and anterograde tracers in striatum, Haber and colleagues uncovered a medio-lateral organization of striatonigrostriatal circuits. Namely, axon terminals from the medial striatum are medially located in the SN and overlap with the cell bodies of neurons that project to the medial and lateral striatum. Axons from the lateral striatum, on the other hand, are laterally located in the SN and overlap with cells that project to the lateral, but not the medial, striatum. Thus, there is a proposed asymmetry in which the medial striatum could influence DA release in the lateral striatum, but the lateral striatum would not influence DA release in the medial striatum. Critically, however, the overlap of axon terminals and cell bodies is neither necessary nor sufficient for the existence of a functional circuit, especially a polysynaptic circuit involving an intermediary GABAergic connection as proposed. Therefore, despite its continuing appeal, the ascending spiral hypothesis rests on weak evidence.

A direct test of the tri-synaptic circuit proposed by the ascending spiral hypothesis has been lacking in part because of technological limitations that prevented selective targeting of projection-specific circuit components. We took advantage of recent developments in transsynaptic tracing (Zingg et al., 2017, 2020) and intersectional genetics (Fenno et al., 2014; Poulin et al., 2018) to solve this problem. Our findings have important implications for *in vivo* DA circuit function and should prompt a reevaluation of the ascending spiral hypothesis.

## RESULTS

### DLS- and DMS-projecting DA neurons are robustly inhibited by SNr

To test whether there is a synaptic basis for the ascending spiral hypothesis and to understand the organization of disinhibitory striatonigrostriatal circuits more generally, we designed a series of experiments using synaptic physiology in combination with carefully targeted optogenetic stimulation. We began by assessing the connectivity of GABAergic SNr cells to DLS- and DMS-projecting DA neurons. Although the SNr is a well-known source of inhibitory input onto SNc DA neurons in general (Tepper and Lee, 2007; Tepper et al., 1995), it was unclear whether the likelihood of receiving GABAergic inputs varied depending on the downstream projection target of the DA neuron.

**Figure 1.**
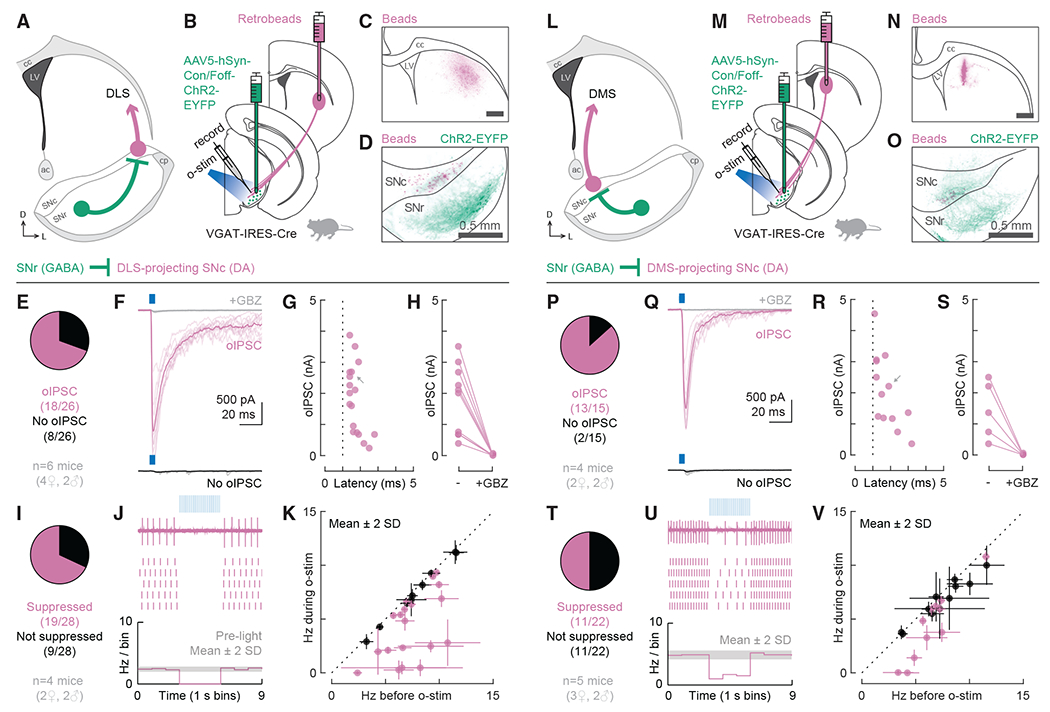
VGAT+ cells in SNr monosynaptically inhibit DLS- and DMS-projecting DA neurons in SNc and suppress their tonic firing (A) Schematic of the tested circuit. Anatomical landmarks: corpus callosum (cc), lateral ventricle (LV), anterior commissure (ac), cerebral peduncle (cp). (B) Experimental design for probing the connection between VGAT+ cells in the SNr and DLS-projecting DA neurons in the SNc. In VGAT-IRES-Cre mice, AAV5-hSyn-Con/Foff-ChR2-EYFP was injected into the SNr to deliver the excitatory opsin ChR2 to VGAT+ cells. Retrobeads were injected into the DLS to label DLS-projecting DA neurons in the SNc for recording. Optogenetic stimulation (o-stim) was delivered via the objective (475 nm, ~10 mW/mm^2^). (C) Distribution of retrobeads (magenta) in a representative striatum slice. Scale bar: 0.5 mm. (D) Distribution of bead-labeled somas (magenta) and ChR2-EYFP-labeled neuropil (green) in a representative midbrain slice. The SNc was outlined based on TH immunolabeling. (E) Proportion of DLS-projecting neurons that did (magenta, n = 18) or did not (black, n = 8) respond to o-stim with an optogenetically evoked inhibitory post-synaptic current (oIPSC; n = 26 cells from 6 mice). (F) Example cells for (E). The oIPSC was absent after gabazine (GBZ) perfusion (gray). Thin lines: individual sweeps. Thick lines: average across sweeps. (G) oIPSC amplitude and onset latency for all responding cells (dotted line = 1 ms). Gray arrow: oIPSC shown in (F). (H) oIPSC amplitude before and after GBZ perfusion for all tested cells. (I) Proportion of DLS-projecting neurons that did (magenta, n = 19) or did not (black, n = 9) have their tonic firing suppressed by o-stim (n = 28 cells from 4 mice). (J) Example recordings for (I). Top: data from a single sweep. Middle: raster plot showing action potentials from 5 sweeps. Bottom: histogram of the average firing rate across all sweeps. The gray shaded area indicates mean ± 2 SDs of the baseline firing rate. (K) Average firing rate during versus before o-stim for all cells from (I) (suppressed cells: magenta; not suppressed: black). Error bars represent ± 2 SDs. Dotted line: unity. (L–V) Same as (A)–(K) but for testing the connection between VGAT+ cells in the SNr and DMS-projecting DA neurons in the SNc. (P–S) 15 cells from 4 mice. (T–V) 22 cells from 5 mice. See also Figure S1.

We labeled projection-defined DA neurons by injecting red retrobeads into the DLS or the DMS (Figure 1). These fluorescently labeled latex beads travel retrogradely from axon terminals to cell bodies and allow for targeted patching of DLS- or DMS-projecting DA neurons in midbrain slices. We are confident that bead-labeled cells are dopaminergic given that (1) bead-labeled cells in the SNc were previously shown to be tyrosine hydroxylase-positive (TH+) (Lerner et al., 2015) and (2) all bead-labeled cells recorded in a loose seal configuration in this study (143/143 cells from 24 mice) had wide action-potential waveforms (total duration >2 ms) characteristic of DA neurons (Grace and Bunney, 1983).

To allow for optogenetic stimulation of GABAergic neurons in the SNr, we injected an adeno-associated virus (AAV) carrying a Cre-dependent channelrhodopsin-2 (ChR2) construct into the SNr of VGAT-IRES-Cre mice. The specific virus used (AAV5-hSyn-Con/Foff-ChR2-EYFP) also contains a feature by which ChR2 expression is turned off by Flp recombinase. In these initial experiments (Figure 1), the Flp-dependent feature is irrelevant. However, it was crucial for later experiments, and so we decided to use the same virus throughout this study.

We began by examining SNr inputs to DLS-projecting DA neurons (Figures 1A and 1B). We verified that all retrobead injections were contained within the DLS (Figures S1A and S1B). As expected from previous findings (Farassat et al., 2019; Haber et al., 2000; Ikemoto, 2007; Lerner et al., 2015), the resulting bead-labeled DLS-projecting DA cells were located in the mid-to-lateral SNc (Figures 1D, S1C, and S1D). We first evaluated the proportion of DLS-projecting DA neurons that were monosynaptically inhibited by GABAergic SNr cells. Our goal was to maximize the detection of inhibitory post-synaptic currents (IPSCs) and minimize false negative results. Therefore, we recorded from bead-labeled cells in whole-cell mode using a high chloride internal solution (E_Cl_ = 0 mV) and held the cells at −70 mV. In addition, we used a pharmacological approach to isolate monosynaptic connections (Petreanu et al., 2009)—we added tetrodotoxin (TTX; 1 μM) to the bath to block action potentials and 4-aminopyridine (4-AP; 100 μM) to boost the neurotransmitter release probability from ChR2-expressing terminals. To isolate inhibitory synapses, we added NBQX (5 μM) and D-AP5 (50 μM) to the bath to block AMPA and NMDA receptor currents, respectively. A 5 ms light pulse (475 nm, ~10 mW/mm^2^) was delivered to the slice to stimulate ChR2-expressing terminals. Under this configuration, we found that 69% (18/26) of the recorded DLS-projecting neurons were monosynaptically inhibited by GABAergic SNr cells (Figures 1E and 1F). The amplitude of the optogenetically evoked IPSCs (oIPSCs) ranged from 0.2 to 3.9 nA (mean ± SD: 1.8 ± 1.1 nA), and the onset latencies were within 5 ms (range: 1.4–2.8 ms; mean ± SD: 1.7 ± 0.4 ms), consistent with the isolation of monosynaptic connections (Figure 1G). For all tested cells, the oIPSC was blocked by the GABA_A_ receptor antagonist gabazine (GBZ; 10 μM; Figure 1H).

These experiments established a robust synaptic connectivity between SNr and DLS-projecting SNc DA neurons, but the measurements were performed under non-physiological conditions (large chloride driving force and high neurotransmitter release probability). Therefore, we additionally wanted to assess whether the observed GABAergic inputs could suppress the tonic firing of DA cells under more physiological conditions. To avoid manipulating intracellular chloride, we recorded from bead-labeled cells in a loose seal configuration. NBQX and D-AP5 were again added to bath but not TTX and 4-AP. For these experiments, we used a 3 s long light train consisting of 5 ms pulses delivered at 20 Hz (475 nm, ~10 mW/mm^2^). A cell was considered inhibited if the light train reduced its firing rate by more than 2 standard deviations (SDs) from the mean (Figures 1J and 1K). Suppression of tonic firing was observed in 68% (19/28) of the recorded DLS-projecting cells (Figure 1I). The percentage of cells whose firing was inhibited by SNr inputs closely matched the percentage in which oIPSCs were observed, arguing that the GABAergic connections detected onto DLS-projecting DA neurons are effective at controlling their firing rates.

We next examined SNr inputs to DMS-projecting DA neurons (Figures 1L and 1M). We verified that all retrobead injections were contained within the DMS (Figures 1N, S1E, and S1F) and that, as expected (Lerner et al., 2015), bead-labeled DMS-projecting DA cells were medially located in the SNc (Figures 1O, S1G, and S1H). Under recording conditions used to isolate monosynaptic inhibitory connections, we found that 87% (13/15) of the recorded DMS-projecting neurons were monosynaptically inhibited by GABAergic SNr cells (Figures 1P and 1Q). The oIPSC amplitude ranged from 0.4 to 4.5 nA (mean ± SD: 2.0 ± 1.2 nA) and the onset latencies were within 5 ms (range: 1.1–3.2 ms; mean ± SD: 1.8 ± 0.7 ms; Figure 1R). For all tested cells, the oIPSC was blocked by GBZ (Figure 1S). Under a loose seal configuration, suppression of tonic firing was observed in 50% (11/22) of the recorded DMS-projecting cells (Figures 1T–1V). In contrast to our findings for DLS-projecting DA neurons, we found a higher percentage of DMS-projecting cells receiving monosynaptic inputs from the SNr (87% versus 69%) but a lower percentage of DMS-projecting cells whose tonic firing was inhibited by the SNr (50% versus 68%).

Collectively, these findings suggest that both DLS- and DMS-projecting DA neurons in the SNc receive robust inhibition from GABAergic SNr cells. Although we did not assess disinhibition directly, such robust inhibition indicates that a decrease in the tonic firing rate of GABAergic SNr cells would be sufficient to disinhibit DLS- and DMS-projecting DA cells. Moreover, our results hint at a dissociation between optogenetically defined synaptic connectivity and effective suppression of tonic firing. Asymmetries in the proportion of connected versus effectively inhibited cells may indicate fundamental differences between subcircuits involving DLS- and DMS-projecting DA neurons.

### Dissection of polysynaptic striatonigrostriatal circuits using a transsynaptic Cre virus and intersectional genetics

The previous experiments assessed two nigrostriatal circuits: SNr→SNc→DLS and SNr→SNc→DMS (Figure 1). We next wanted to layer on to our assessment of these circuits the contributions of striatal inputs to the SNr, which would allow either for striatal neurons to control disinhibition of their own dopaminergic input (through closed loops such as DLS→SNr→SNc→DLS) or for one striatal region to regulate dopaminergic transmission in a neighboring region (e.g., DMS→SNr→SNc→DLS) as proposed in the ascending spiral hypothesis (Haber et al., 2000; Yin and Knowlton, 2006). While hypotheses about DA neuron disinhibition through striatonigrostriatal circuits are often incorporated into theory (e.g., Lüscher et al., 2020), the difficulty of tracing synaptic connectivity through a polysynaptic circuit has impeded their testability. Therefore, hypotheses about the structure and function of these circuits have remained highly speculative. We realized that new anterograde tracing (Zingg et al., 2017, 2020) and combinatorial targeting tools (Fenno et al., 2014) would allow highly specific tests of the structure and function of striatonigrostriatal circuits.

**Figure 2.**
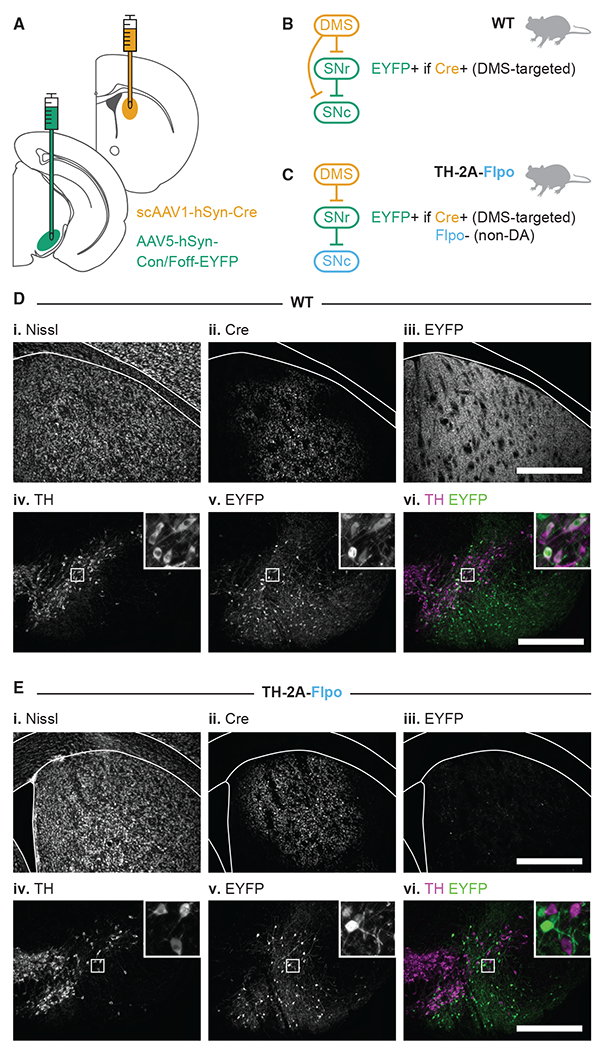
Viral strategy used for polysynaptic circuit dissection (A) Experimental design for labeling DMS-targeted non-dopaminergic neurons in the SNr. scAAV1-hSyn-Cre injected into the DMS moves trans-synaptically in the anterograde direction to deliver Cre to DMS-targeted neurons. AAV5-hSyn-Con/Foff-EYFP is injected into the SNr to deliver EYFP to cells that are both Cre+ and Flp−. (B) Schematic of the resulting EYFP labeling in a WT mouse (all cells are Flp−; both GABA and DA cells may be Cre+). (C) Schematic of the resulting EYFP labeling in a TH-2A-Flpo mouse (DA cells are Flp+; only DMS-targeted, non-DA cells are Flp− and Cre+). (D and E) Example histology from the striatum (top row) and the SN (bottom row) after injections in WT (D) and TH-2A-Flpo (E) mice. Scale bar: 0.5 mm. See also Figure S2.

To label SNr cells by their striatal inputs, we used scAAV1-hSyn-Cre as a transsynaptictranssynaptic anterograde Cre vector (Zingg et al., 2020). When injected into the DLS or the DMS, this virus will transduce SPNs at the injection site and the post-synaptic targets of these SPNs throughout the brain. Thus, cells that receive a monosynaptic input from the DLS or the DMS will also carry Cre. We refer to these anterogradely labeled cells as “DLS-targeted” and “DMS-targeted,” respectively. Before continuing our electrophysiology experiments, we examined the resulting histology in the striatum and the substantia nigra (SN) after injection of scAAV1-hSyn-Cre into the striatum (Figure 2).

First, we injected wild-type (WT) mice (Figure 2B). We verified that scAAV1-hSyn-Cre did not lesion the striatum, as evidenced by healthy Nissl staining (Figure 2Di) and observed that Cre expression was restricted to the targeted region (Figure 2Dii). Next, we looked for Cre expression in the SNr. To do so, we injected a Cre-dependent EYFP construct (AAV5-hSyn-Con/Foff-EYFP) into the SNr. EYFP+ cells were observed in the SNr (Figure 2Dv), but EYFP+ fibers were also observed in the striatum (Figure 2Diii). GABAergic SNr cells receive monosynaptic inputs from the striatum but do not project directly to the striatum, whereas dopaminergic SNc neurons do both (Evans et al., 2020; Lerner et al., 2015; Matsuda et al., 2009; Watabe-Uchida et al., 2012; Zingg et al., 2020). Thus, EYFP+ fibers observed within the striatum indicate that DA neurons received Cre. Indeed, after immunostaining for the DA marker TH, we confirmed that EYFP-labeled cells in the SN included both TH− and TH+ cells (Figure 2Dvi). It is also possible that some DA neurons received Cre through unintended retrograde movement of the transsynaptic Cre virus (Hollis et al., 2008; Zingg et al., 2017, 2020). However, any retrograde movement of the virus does not affect the labeling of SNr neurons since these cells do not project to striatum (McElvain et al., 2021).

While not surprising, the finding that SNc DA neurons are labeled with Cre by injection of scAAV1-hSyn-Cre in the striatum presented a problem for our experimental design, which required that we limit ChR2 expression to GABAergic SNr neurons. Therefore, we used an intersectional Cre/Flp recombinase expression strategy to exclude expression of EYFP/ChR2 from DA neurons. Namely, we injected scAAV1-hSyn-Cre into the DMS of TH-2A-Flpo mice, which express Flp recombinase in DA neurons (Poulin et al., 2018). We then injected the same EYFP virus as above (AAV5-hSyn-Con/Foff-EYFP) into the SNr. The Con/Foff construct allows expression of EYFP in cells that express Cre but not Flp. Therefore, we could positively label non-DA SNr neurons identified as receiving input from a particular striatal subregion (Figure 2C). Using this strategy, we did not find evidence of overlapping EYFP and TH expression in the SN (Figure 2E–vi). In addition, we did not observe EYFP+fibers in the striatum (Figure 2Eiii). The success of this strategy is more easily visualized with an EYFP virus, which labels the cytoplasm of neurons, but was equally successful when we used a ChR2 virus (AAV5-hSyn-Con/Foff-ChR2-EYFP; Figure S2).

In sum, we can deliver ChR2 to DMS- and DLS-targeted non-DA cells in the SN with two viral injections in a TH-2A-Flpo mouse: a transsynaptic anterograde Cre virus in the striatum (DMS or DLS) and a Con/Foff-ChR2 virus in the SNr.

### Characterization of closed striatonigrostriatal loops

By combining retrobead injections in striatum with our viral strategy in TH-2A-Flpo mice, we could investigate the structure and function of multiple striatonigrostriatal circuits. Because basal ganglia circuits are thought to operate primarily in parallel closed loops (Alexander et al., 1986; Haber et al., 2000; Lee et al., 2020; Mandelbaum et al., 2019; Yin and Knowlton, 2006), we began by testing closed striatonigrostriatal loops through which the DLS and the DMS could regulate their own dopaminergic drive.

**Figure 3.**
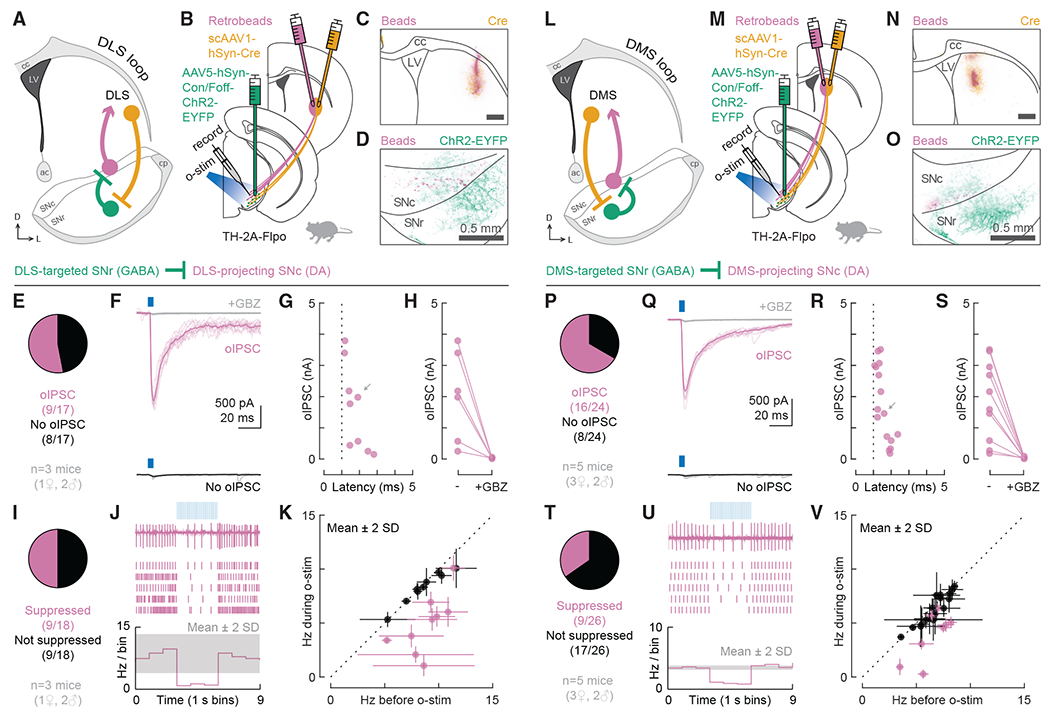
DLS- and DMS-targeted GABAergic cells in SNr monosynaptically inhibit DLS- and DMS-projecting DA neurons in SNc, respectively, and suppress their tonic firing (A) Schematic of the DLS loop. (B) Experimental design for probing the connection between DLS-targeted GABAergic cells in the SNr and DLS-projecting DA neurons in the SNc. In TH-2A-Flpo mice, scAAV1-hSyn-Cre was injected into the DLS to label DLS-targeted cells with Cre. AAV5-hSyn-Con/Foff-ChR2-EYFP was injected into the SNr to deliver ChR2 to cells carrying Cre but not Flp. Retrobeads were injected into the DLS to label DLS-projecting DA neurons in the SNc for recording. (C) Distribution of retrobeads (magenta) and Cre (yellow) in a representative striatum slice. Scale bar: 0.5 mm. (D) Distribution of bead-labeled somas (magenta) and ChR2-EYFP-labeled neuropil (green) in a representative midbrain slice. (E) Proportion of DLS-projecting neurons that did (magenta, n = 9) or did not (black, n = 8) respond to o-stim with an oIPSC (n = 17 cells from 3 mice). (F) Example cells for (E). (G) oIPSC amplitude and onset latency for all responding cells. Gray arrow: oIPSC shown in (F). (H) oIPSC amplitude before and after GBZ perfusion. (I) Proportion of DLS-projecting neurons that did (magenta, n = 9) or did not (black, n = 9) have their tonic firing suppressed by o-stim (n = 18 cells from 3 mice). (J) Example cell for (I). Top: data from a single sweep. Middle: raster plot showing action potentials from 5 sweeps. Bottom: average firing rate across all sweeps. The gray shaded area indicates mean ± 2 SDs of the baseline firing rate. (K) Average firing rate during versus before o-stim for all cells from (I). Error bars represent ± 2 SDs. Dotted line: unity. (L–V) Same as (A)–(K) but for testing the DMS loop. (P–S) 24 cells from 5 mice. (T–V) 26 cells from 5 mice. See also Figure S3.

To test a closed DLS loop (Figure 3A), we injected both the transsynaptic Cre virus (scAAV1-hSyn-Cre) and red retrobeads into the DLS of TH-2A-Flpo mice. We also injected AAV5-hSyn-Con/Foff-ChR2-EYFP into the SNr. With this design, we could record from bead-labeled DLS-projecting DA neurons in the SNc while optogenetically stimulating DLS-targeted GABAergic neurons in the SNr (Figure 3B). We verified that all DLS injections were contained within the DLS (Figure 3C, S3A, and S3B). We also observed that both bead-labeled somas and ChR2-EYFP+ neuropil were located in the mid-lateral SN (Figure 3D). Under recording conditions used to isolate monosynaptic inhibitory connections, we found that 53% (9/17) of the recorded DLS-projecting neurons were monosynaptically inhibited by DLS-targeted GABAergic cells in the SNr (Figure 3E and 3F). The oIPSC amplitude ranged from 0.2 to 3.8 nA (mean ± SD: 1.6 ± 1.3 nA), and the onset latencies were 1.2–2.8 ms (mean ± SD: 1.7 ± 0.5 ms; Figure 3G). For all tested cells, the oIPSC was blocked by GBZ (Figure 3H). Under a loose seal configuration, suppression of tonic firing was observed in 50% (9/18) of the recorded DLS-projecting cells (Figures 3I–3K). The percentage of cells whose firing was suppressed closely matched the percentage in which oIPSCs were observed (50% versus 53%), recapitulating the correlation between effective inhibition and synaptic connectivity observed for DLS-projecting DA neurons previously (Figures 1E–1K, 68% versus 69%).

We next examined a closed DMS loop by injecting both the transsynaptic Cre virus (scAAV1-hSyn-Cre) and red retrobeads into the DMS of TH-2A-Flpo mice and injecting AAV5-hSyn-Con/Foff-ChR2-EYFP into the SNr. We recorded from bead-labeled DMS-projecting DA neurons in the SNc while optogenetically stimulating DMS-targeted GABAergic neurons in the SNr (Figures 3L and 3M). We verified that all DMS injections were contained within the DMS (Figures 3N, S3E, and S3F). We also observed that both bead-labeled somas and ChR2-EYFP+ neuropil were medially located in the SN (Figure 3O). Under recording conditions used to isolate monosynaptic inhibitory connections, we found that 67% (16/24) of the recorded DMS-projecting neurons were monosynaptically inhibited by DMS-targeted GABAergic cells in the SNr (Figures 3P and 3Q). The oIPSC amplitude ranged from 0.2 to 3.5 nA (mean ± SD: 1.8 ± 1.2 nA), and the onset latencies were 1.1–2.4 ms (mean ± SD: 1.6 ± 0.4 ms; Figure 3R). For all tested cells, the oIPSC was blocked by GBZ (Figure 3S). Under a loose seal configuration, suppression of tonic firing was observed in 35% (9/26) of the recorded DMS-projecting cells (Figures 3T–3V). The percentage of cells whose firing was inhibited was approximately half of the percentage in which oIPSCs were observed (35% versus 67%), corroborating the dissociation between effective inhibition and synaptic connectivity observed for DMS-projecting DA neurons previously (Figures 1P–1V, 50% versus 87%).

Collectively, these findings confirm the existence of closed striatonigrostriatal loops through which the DLS and the DMS could alter their own dopaminergic drive via inhibition of GABAergic cells in the SNr and disinhibition of DA cells in the SNc. Moreover, our findings suggest that the DLS would be more effective at such disinhibition than the DMS.

### Open spiral striatonigrostriatal circuits are unlikely to support robust DA disinhibition

**Figure 4.**
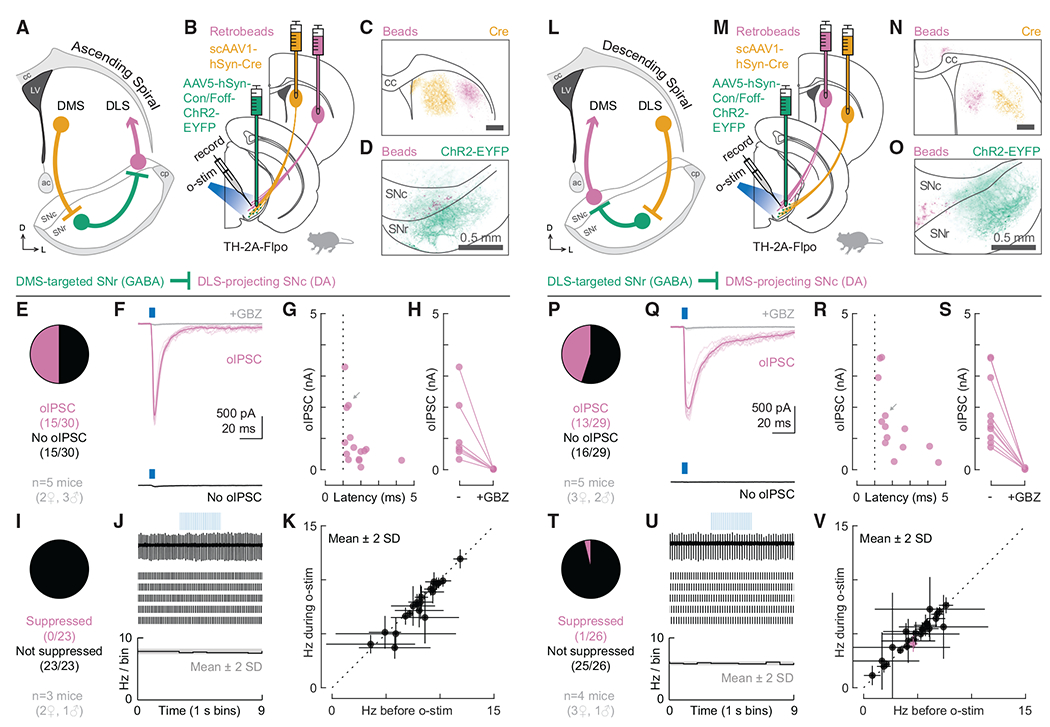
DMS- and DLS-targeted GABAergic cells in SNr monosynaptically inhibit DLS- and DMS-projecting DA neurons in SNc, respectively, but do not suppress their tonic firing (A) Schematic of the ascending spiral. (B) Experimental design for probing the connection between DMS-targeted GABAergic cells in the SNr and DLS-projecting DA neurons in the SNc. (C) Distribution of retrobeads (magenta) and Cre (yellow) in a representative striatum slice. Scale bar: 0.5 mm. (D) Distribution of bead-labeled somas (magenta) and ChR2-EYFP-labeled neuropil (green) in a representative midbrain slice. (E) Proportion of DLS-projecting neurons that did (magenta, n = 15) or did not (black, n = 15) respond to o-stim with an oIPSC (n = 30 cells from 5 mice). (F) Example cells for (E). (G) oIPSC amplitude and onset latency for all responding cells. Gray arrow: oIPSC shown in (F). (H) oIPSC amplitude before and after GBZ perfusion. (I) Proportion of DLS-projecting neurons that did (magenta, n = 0)or did not (black, n = 23) have their tonic firing suppressed by o-stim (n = 23 cells from 3 mice). (J) Example cell for (I). Top: data from a single sweep. Middle: raster plot showing action potentials from 5 sweeps. Bottom: average firing rate across all sweeps. The gray shaded area indicates mean ± 2 SDs of the baseline firing rate. (K) Average firing rate during versus before o-stim for all cells from (I). Error bars represent ± 2 SDs. Dotted line: unity. (L–V) Same as (A)–(K) but for testing the descending spiral. (P–S) 29 cells from 5 mice. (T–V) 26 cells from 4 mice. See also Figure S4.

After employing our experimental strategy to test closed striatonigrostriatal loops, we used a similar approach to test open-loop spiral circuits, beginning with the ascending spiral circuit (DMS→SNr→SNc→DLS). We injected the transsynaptic Cre virus (scAAV1-hSyn-Cre) into the DMS and red retrobeads into the DLS of TH-2A-Flpo mice (Figure 4A). We also injected AAV5-hSyn-Con/Foff-ChR2-EYFP into the SNr. With this design, we could record from bead-labeled DLS-projecting DA neurons in the SNc while optogenetically stimulating DMS-targeted GABAergic neurons in the SNr (Figure 4B). We verified that all injections in the striatum were contained within their target areas (Figure 4C, S4A, and S4B). We also observed an overlap of bead-labeled cells and ChR2-EYFP+ neuropil in the SN (Figure 4D), consistent with the predictions of the ascending spiral hypothesis (Haber et al., 2000). Under recording conditions used to isolate monosynaptic inhibitory connections, we found that 50% (15/30) of the recorded DLS-projecting neurons were monosynaptically inhibited by DMS-targeted GABAergic cells in the SNr (Figures 4E and 4F). The oIPSC amplitude ranged from 0.1 to 3.3 nA (mean ± SD: 0.9 ± 0.9 nA), and the onset latencies were 1.1–4.3 ms (mean ± SD: 1.8 ± 0.8 ms; Figure 4G). For all tested cells, the oIPSC was blocked by GBZ (Figure 4H). Under a loose seal configuration, however, suppression of tonic firing was not observed in any of the recorded DLS-projecting cells (0/23; Figures 4I–4K). The striking mismatch between the percentage of cells whose firing was inhibited and the percentage in which oIPSCs were observed was unexpected and in stark contrast to the nearly perfect match between synaptic connectivity and effective inhibition for DLS-projecting cells in our previous experiments (Figures 1A–1K and 3A–3K). Our findings suggest that there is a fundamental difference between the closed DLS loop and the ascending spiral connecting the DMS to the DLS. Although synaptic connections exist at roughly similar rates in the two circuits (53% versus 50%), the ability of these circuits to control the tonic firing of DA neurons is remarkably different (50% versus 0%).

In previous work establishing the ascending spiral hypothesis, a lack of overlap between axons from lateral striatum and the cell bodies of SN neurons projecting to the medial striatum was noted (Haber et al., 2000). This result led to the prediction that there is limited connectivity in a descending spiral (DLS→SNr→SNc→DMS), yet this prediction has not been tested. Indeed, such overlap is not necessary for the existence of a functional polysynaptic circuit. To examine the descending spiral circuit (Figure 4L), we injected the transsynaptic Cre virus (scAAV1-hSyn-Cre) into the DLS and red retrobeads into the DMS of TH-2A-Flpo mice. We also injected AAV5-hSyn-Con/Foff-ChR2-EYFP into the SNr. With this design, we could record from bead-labeled DMS-projecting DA neurons in the SNc while optogenetically stimulating DLS-targeted GABAergic neurons in the SNr (Figure 4M). We verified that all injections in the striatum were contained within their target areas (Figures 4N, S4E, and S4F). We observed poor overlap of bead-labeled cells and ChR2-EYFP+ neuropil in SN (Figure 4O), consistent with the predictions of Haber and colleagues work in non-human primates (Haber et al., 2000). However, despite the lack of overlap, we found that 45% (13/29) of the recorded DMS-projecting neurons were monosynaptically inhibited by DLS-targeted GABAergic cells in the SNr (Figures 4P and 4Q). The oIPSC amplitude ranged from 0.2 to 3.6 nA (mean ± SD: 1.5 ± 1.1 nA), and the onset latencies were 1.2–4.6 ms (mean ± SD: 2.1 ± 1.1 ms; Figure 4R). For all tested cells, the oIPSC was blocked by GBZ (Figure 4S). The connectivity we observed was surprising. However, we did not observe inhibition of tonic firing through these synaptic connections. Under a loose seal configuration, suppression of tonic firing was observed in only 4% (1/26) of the recorded DMS-projecting cells (Figures 4T–4V). The striking mismatch between synaptic connectivity and inhibition of tonic firing was once again unexpected but not as surprising, given that some mismatch was previously observed for DMS-projecting cells (Figures 1P–1V and 3P–3V). Together, our results from testing the ascending and descending spiral circuits suggest that these circuits are unlikely to support robust DA neuron disinhibition, at least in naive mice.

### Strong GABAergic SNr inputs onto DA neurons do not predict inhibition of tonic firing

**Figure 5.**
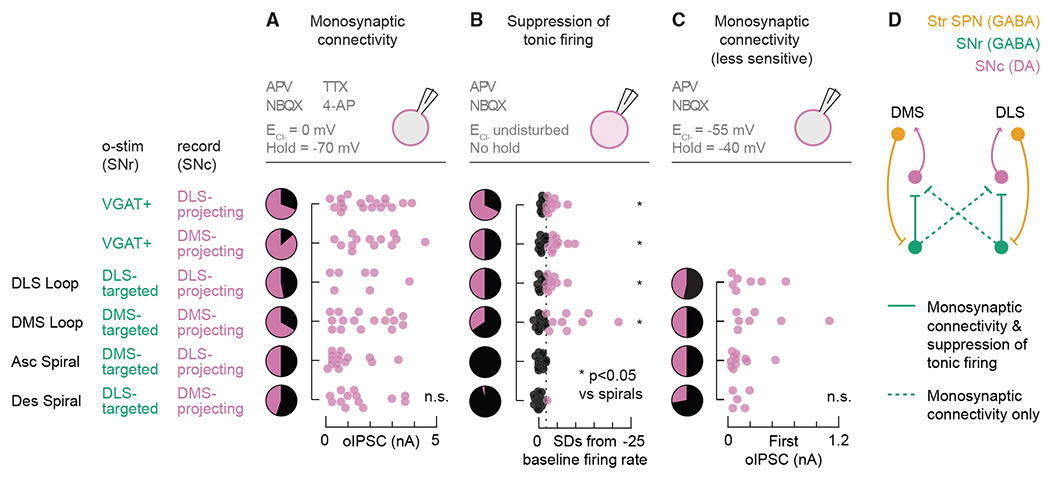
Closed loops are supported by monosynaptic connectivity and suppression of tonic firing, while open spirals are supported by monosynaptic connectivity only (A–C)Top: recording configuration. Pie charts: proportion of bead-labeled neurons that did (magenta) or did not (black) respond to o-stim. Scatterplots: (A and C) oIPSC amplitude or (B) change in tonic firing rate from baseline. Dotted line: −2 SDs. n.s., not significant. *p < 0.05 versus ascending spiral and versus descending spiral. (A and B) Data reproduced from Figures 1, 3, and 4. (C) For cell and mouse numbers, see Figure 6. (D) Circuit diagram supported by the data.

In both open- and closed-loop striatonigrostriatal circuits, we observed robust GABAergic connectivity from SNr neurons onto DA SNc neurons, mediated by GABA_A_ receptor transmission. Given this connectivity, and the fact that the amplitude of the recorded oIPSCs was similar in all circuit configurations (Figure 5A), we expected to observe similar rates of suppression of DA neuron firing across conditions. Surprisingly, we found instead that the tonic firing of DA neurons was clearly inhibited in closed loops but not open spirals (Figure 5B).

The dissociation we observed between monosynaptic connectivity and firing suppression could be explained by technical differences between the testing conditions. During the detection of oIPSCs, we used 4-AP to boost neurotransmitter release probability from GABAergic SNr cells when action potentials were blocked by TTX, but a more physiological release probability was preserved during loose seal recordings. 4-AP could have masked oIPSC amplitude differences between low and high release-probability synapses. Furthermore, the use of a high chloride internal during the detection of oIPSCs often resulted in large (>1 nA) currents, which could have impaired our ability to voltage clamp. Thus, we repeated our monosynaptic connectivity experiments in the absence of TTX and 4-AP and used an internal solution with a lower chloride concentration to better mimic the physiological chloride reversal potential. Under these conditions, we observed smaller oIPSCs (most <1 nA), yet we still found no differences in oIPSC amplitudes between circuit configurations (Figure 5C). We also reproduced the connection probabilities previously observed for DLS-projecting cells (47% versus 53% for DLS loop and 50% versus 50% for ascending spiral) but found lower connection probabilities for DMS-projecting cells (50% versus 67% for DMS loop and 28% versus 45% for descending spiral). Under these recording conditions, monosynaptic connectivity rates once again perfectly predict firing suppression rates for the DLS loop, but the dissociation for the other circuits–which is particularly stark for the ascending spiral–remains unexplained. Collectively, these findings support a model in which only closed striatonigrostriatal loops induce strong firing suppression, although latent functional connectivity is present in open spirals (Figure 5D).

### Differences between closed loops and open spirals are not explained by differences in short-term plasticity

One explanation for the dissociation between connectivity and firing suppression could be short-term plasticity. Given that firing suppression was assessed with a light train containing 60 pulses over 3 s, it could be that SNr→SNc synapses in open spirals are prominent initially but robustly depressing during the light train. If so, inhibition would not be sustained over the course of seconds.

**Figure 6.**
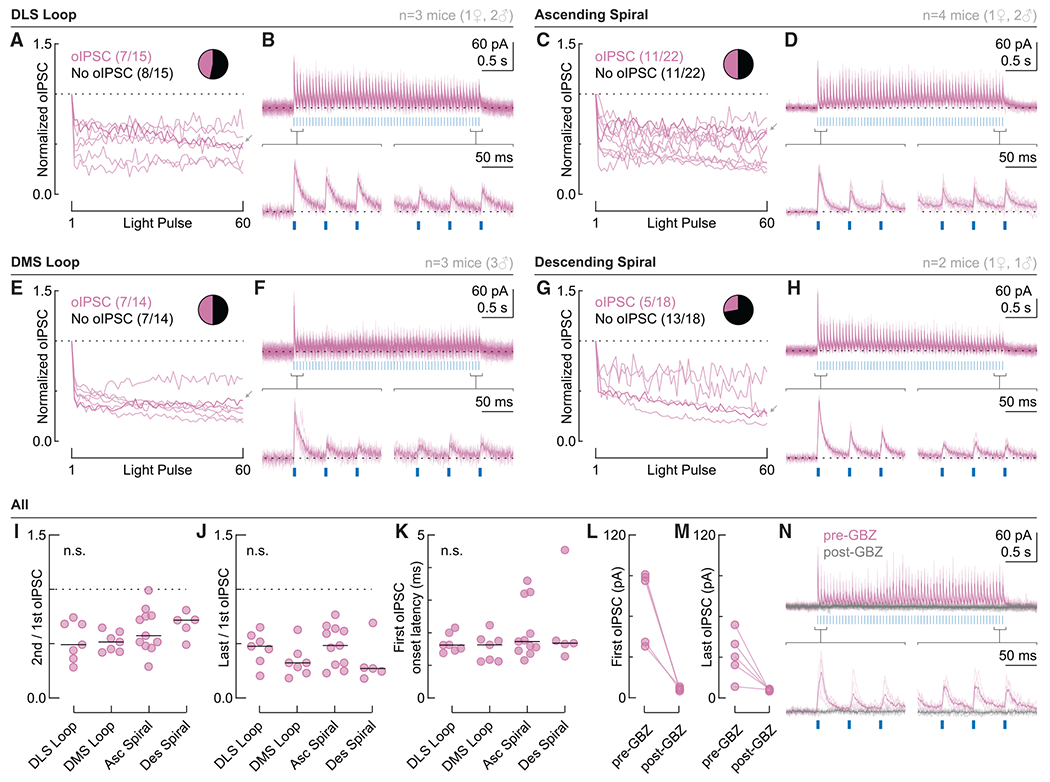
Pre-synaptic release probability does not explain differences between closed loops and open spirals (A and B) DLS loop dataset. (A) Normalized oIPSC amplitude for bead-labeled neurons that responded to o-stim (20 Hz, 3 s; n = 7 cells from 3 mice). Dotted line: 1. Insert: proportion of bead-labeled neurons that did (magenta, n = 7) or did not (black, n = 8) respond to o-stim. Gray arrow: example cell shown in (B). (B) Example cell with a zoom in of the first and last three oIPSCs. Thin lines: individual sweeps. Thick lines: average across sweeps. Dotted line: baseline. (C and D) Same as (A) and (B) but for the ascending spiral (n = 22 cells–11 responding to o-stim–from 4 mice). (E and F) Same as (A) and (B) but for the DMS loop (n = 14 cells–7 responding to o-stim–from 3 mice). (G and H) Same as (A) and (B) but for the descending spiral (n = 18 cells–5 responding to o-stim–from 2 mice). (I) Ratio between the second and first oIPSC. (J) Ratio between the last and first oIPSC. (K) Onset latency of the first oIPSC. Black bars in (I)–(K) indicate the median. n.s., not significant. (L and M) First (L) and last (M) oIPSC amplitude before and after GBZ perfusion for all tested cells. (N) Example recording before (magenta) and after (gray) GBZ perfusion.

To test this possibility, we stimulated the cells shown in Figure 5C with the same optogenetic stimulation (o-stim) used for loose seal recordings (3 s, 20 Hz) and measured the amplitude of all oIPSCs relative to the first. We found that cells in all circuit configurations showed modest short-term depression (Figures 6A–6H). A few cells displayed delayed facilitation relative to the second oIPSC (see Figure 6C, for example), but there were no clear differences between closed loops and open spirals. The paired-pulse ratio between the second and first oIPSC was not significantly different between groups (Figure 6I) and neither was the ratio between the last and first oIPSC (Figure 6J). Despite the lack of TTX in the bath, the detected oIPSCs are likely to be monosynaptic given their onset latency (Figure 6K). All cells tested displayed oIPSCs mediated entirely by GABA_A_ receptors (Figures 6L–6N). Collectively, these data show that the observed differences between closed loops and open spirals are not due to differences in short-term plasticity.

## DISCUSSION

### Evidence for and against the ascending spiral hypothesis

We tested multiple striatonigrostriatal loops connecting two striatal subregions (DMS and DLS) via the SNr and the SNc (i.e., DMS→DLS→SNr→SNc→DMS/DLS). These loops have the potential to transform activity in a striatal subregion into DA release in the same or neighboring region of the striatum by disinhibiting dopaminergic neurons in the SNc. We were particularly interested in testing the predictions of the ascending spiral hypothesis, which argues that an open-loop striatonigrostriatal circuit permits the progressive disinhibition of DA neurons in a unidirectional, ascending (medio-lateral) direction (Haber et al., 2000). We focused on circuits involving the dorsal striatum given that the ascending spiral hypothesis is frequently invoked to explain changes in the DMS and the DLS that occur over the course of extended training, as animals become proficient in motor skill tasks or transition from goal-directed to habitual behavior.

Our data provide evidence both for and against the ascending spiral hypothesis. They support the existence of a DMS→SNr→SNc→DLS circuit but challenge the prediction that this circuit alone can support disinhibition in DA neurons. Instead, our data suggest that closed striatonigrostriatal loops (i.e., DMS→SNr→SNc→DMS and DLS→SNr→SNc→DLS) are better suited to support disinhibition. Our findings are complemented by findings in ventral striatal circuits, which similarly suggest that disinhibition operates primarily in closed loops (Yang et al., 2018). Our findings further diverge from the ascending spiral hypothesis by documenting the existence of a descending spiral (DLS→SNr→SNc→DMS) of approximately equal strength to the ascending spiral, challenging the claim of unidirectional information flow.

These results are important because previous anatomical findings about the topography of striatonigrostriatal circuits (Haber et al., 2000) have inspired the field to interpret behavioral and neural activity findings under the framework of an ascending spiral (Lerner, 2020; Lüscher et al., 2020; Yin and Knowlton, 2006). Indeed, the sequential recruitment of the DMS and the DLS during motor skill learning and habit formation fits nicely with the ascending spiral hypothesis (Gremel and Costa, 2013; Thorn et al., 2010; Yin et al., 2009); so does the dependence of habit formation on DA projections to the DLS (Faure et al., 2005) and the increasing recruitment of DLS DA activity with drug use (Belin and Everitt, 2008; Willuhn et al., 2012). In addition, modeling studies point to striatonigral circuits in the form of Str→SNr→SNc as a robust means of disinhibition and burst firing in dopaminergic neurons (Lobb et al., 2011). Although no direct evidence exists for impaired DA release in the DLS following DMS lesions, ventromedial striatum (VMS) lesions are reported to impair DLS DA release (Willuhn et al., 2012).

While these previous findings are consistent with the ascending spiral hypothesis, direct evidence for a continuous polysynaptic circuit connecting DMS→SNr→SNc→DLS was lacking, and other findings do not fit. For instance, if repeated activation of the DMS is required to elicit DA release in the DLS and drive motor learning and habit formation, one would expect DMS lesions to hinder these processes, but that is not the case. Instead, DMS lesions do not prevent motor skill learning (Yin et al., 2009) and are in fact reported to accelerate habit formation (Gremel and Costa, 2013; Yin et al., 2004, 2005a, 2005b). It is also hard to reconcile the slow time course of habit formation and the associated changes in the DLS (days to weeks) with a tri-synaptic circuit theoretically capable of regulating DA release in the DLS within tens of milliseconds. One possible explanation is that a disinhibitory ascending spiral circuit is not fully functional in naive animals but develops slowly during training. The latent synaptic connections we observed in the DMS→SNr→SNc→DLS circuit could undergo plasticity and/or regulate the plasticity of other inputs onto DA neurons over the course of training even if they do not regulate DA neuron firing in naive mice. Therefore, adjustments to the ascending spiral hypothesis that incorporate experience-dependent plasticity may be warranted.

### Potential mechanisms for the dissociation between connectivity and firing-rate modulation

Presynaptic short-term plasticity mechanisms did not explain the dissociation we observed (Figure 6). Therefore, we suspect that a postsynaptic mechanism is involved. DA neurons, which do not express the chloride extruder potassium-chloride cotransporter 2 (KCC2), have a weakly hyperpolarizing chloride reversal potential (Gulácsi et al., 2003). Therefore, inhibition through GABA_A_ receptor activity is primarily due to shunting inhibition and will be less effective at regulating firing rates if synapses are located far from the action-potential-generating mechanisms of the DA cell. In other words, one might expect lower rates of firing modulation compared with rates of monosynaptic connectivity if synapses are located on distal dendrites. We hypothesize that preferential targeting of distal DA neuron dendrites is the key difference between open-spiral and closed-loop SNr→SNc synapses, a topic for future study.

Compartmentalization of synaptic inputs has been previously reported for midbrain DA neurons as has heterogeneity in intrinsic properties (Crittenden et al., 2016; Evans et al., 2017, 2020; Farassat et al., 2019; Lammel et al., 2008, 2011; Tarfa et al., 2017). Notably, striosome SPNs target the distal SNr dendrite of SNc DA neurons, in so-called striosome-dendron bouquets (Crittenden et al., 2016), while neurons of the globus pallidus external (GPe) segment target the soma and proximal dendrites of DA neurons (Evans et al., 2020). Moreover, striosomes target ventral tier DA neurons, which have a prominent sag current and after depolarization that support rebound firing (Evans et al., 2017, 2020). Interactions between intrinsic properties and preferential targeting could explain the differences we observed between closed and open loops. Additional layers of synaptic input integration would be possible if, like hippocampal and cortical neurons, DA cells maintain a compartmentalized responsiveness to GABAergic inputs due to subcellular variance in intracellular chloride (Khirug et al., 2008; Rahmati et al., 2021).

Activation of slow inhibitory conductances through GABA_B_ receptors or other G-protein-coupled receptors (GPCRs) might also explain the dissociation we observed. However, our data suggest that this is not the case. In the dataset shown in Figure 6, we identified a slow hyperpolarizing current in a subset of cells, but the relative number of cells with this current was comparable across circuit configurations and therefore unlikely to explain the differences between closed and open loops (DLS loop: 3/7 cells; DMS loop: 4/7; ascending spiral: 7/11; descending spiral: 3/5). Furthermore, this slow current is dependent on GABA_A_ receptors, given that it is sensitive to GBZ (see example in Figure 6N).

### Alternatives to the ascending spiral hypothesis

The ascending spiral hypothesis as formulated by Haber and colleagues is not the only means by which striatal subregions could influence each other. For example, the VMS can modulate DLS activity via a long polysynaptic loop through the SNr, thalamus, and motor cortex (Aoki et al., 2019), bypassing not only DMS but also DA neurons. Other mechanisms might exist through lateral inhibition among SPNs (Burke et al., 2017), striatal interneuron networks (Cai and Ford, 2018; Dorst et al., 2020; Fino et al., 2018; Holly et al., 2019; Xu et al., 2015), modulation of DA axon terminals (Kramer et al., 2020; Liu et al., 2021; Mohebi et al., 2019), or striatal astrocyte networks (Khakh, 2019). Thus, even if the ascending spiral circuit for DMS-DLS communication through the control of DA neuron activity is weak, other circuits may instead support information transfer between the DMS and the DLS.

### Balancing striatal inhibition and disinhibition of DA neurons

Given the findings described here regarding the indirect connections between the striatum and the SNc via the SNr and previous research on the direct connections between the striatum and the SNc, it is hard to predict which patterns of striatal activity would support the disinhibition of DA neurons *in vivo*. Multiple rabiestracing studies have characterized the monosynaptic inputs onto projection-defined DA neurons and identified the striatum as a major source of direct inhibition to DA cells (Lerner et al., 2015; Menegas et al., 2015; Watabe-Uchida et al., 2012). However, these direct connections were excluded from computational models of striatonigrostriatal circuits that predicted disinhibition of DA neurons following striatal activation (Lobb et al., 2011). Lerner and colleagues further dissected these direct striatonigrostriatal circuits with slice electrophysiology and found that the DMS preferentially targets DMS-projecting DA neurons, while the DLS targets both DMS- and DLS-projecting DA neurons. Thus, monosynaptic connections between the striatum and the SNc support the existence of closed loops (DMS→SNc→DMS and DLS→SNc→DLS), as well as a descending circuit (DLS→SNc→DMS). Work in ventral striatal circuits also draws attention to the role of direct inhibition of DA neurons by striatal inputs, which can be mediated by GABA_B_ as well as GABA_A_ receptors (Yang et al., 2018). Further investigation is required to compare the relative strength of direct and indirect striatonigrostriatal circuits on the activity of DA neurons and test the conditions that favor disinhibition over inhibition *in vivo*.

Finally, it is possible that the balance between inhibition and disinhibition of DA neurons is altered by training, either by synaptic plasticity or by the recruitment of additional circuits during learning. We and others have observed that the *in vivo* patterns of DA axon activity and DA release in the DMS and the DLS change with training (Hamid et al., 2021; Seiler et al., 2022; Willuhn et al., 2012). The reasons for training-induced changes in DA signaling are not yet clear, but with the approaches developed here and with additional innovations to adapt them for *in vivo* investigations, we can begin to rigorously address this hypothesis and advance our mechanistic understanding of the complex process of habit formation.

### Limitations of the study

Two technical caveats could result in underestimation of the connectivity probabilities reported here: (1) incomplete penetrance of our labeling methods and (2) severing of the distal dendrites of DA neurons in midbrain slices. Although our labeling methods are not 100% penetrant, any underestimation due to this caveat should affect all tested circuits similarly since we used the same viruses and retrobeads in all experiments. The severing of distal dendrites, on the other hand, could disproportionately affect some circuit configurations. The SN has a complex three-dimensional structure that is not fully preserved in coronal slices (Gerfen et al., 1987; Maurin et al., 1999). If a particular subpopulation of SNr cells targets the distal dendrites of DA neurons, then this connection is more likely to be underestimated. Additionally, if DA neurons projecting to the DLS or the DMS belong predominantly to ventral-tier SNc and have a prominent distal dendrite in the SNr (Gerfen et al., 1987), connections onto these cells are also more likely to be underestimated. Fortunately, these caveats do not seem to significantly bias our results, given that oIPSCs of similar amplitudes were detected in all circuit configurations (Figure 5). In addition, we assessed synaptic connectivity and effects on tonic firing in slices from the same mice. DA cells that were not suppressed by o-stim were often located adjacent to DA cells that exhibited robust oIPSCs. Hence, the dissociation between synaptic connectivity and effective inhibition we report is not due to variability in slicing and/or ChR2 expression across animals. We were also careful to sample bead-labeled cells across the entire volume of the SN to avoid any biases regarding the location of DA neurons (Figures S1, S3, and S4). We did not observe any correlations between cell location and likelihood of connection for any of the tested circuits.

## STAR★METHODS

### RESOURCE AVAILABILITY

#### Lead contact

Further information and requests for resources and reagents should be directed to and will be fulfilled by the lead contact, Talia N. Lerner (talia.lerner@northwestern.edu).

#### Materials availability

This study did not generate new unique reagents.

#### Data and code availability

- All data reported in this paper will be shared by the lead contact upon request.
- This paper does not report original code.
- Any additional information required to reanalyze the data reported in this paper is available from the lead contact upon request.

### EXPERIMENTAL MODEL AND SUBJECT DETAILS

#### Mice

Male and female C57BL/6J mice were group housed under a conventional 12:12 h light/dark cycle with ad libitum access to food and water. The VGAT-IRES-Cre knock-in strain was obtained from The Jackson Laboratory (Jackson Stock #028862) and the TH-2A-Flpo line was a gift from Dr. Awatramani (Poulin et al., 2018; MMRRC Stock #050618-MU). Animals were bred in-house, and only heterozygous transgenic mice were used for experiments. WT mice used in Figures 2 and S2 were Flp mice from our TH-2A-Flpo breeding. Littermates were randomly assigned to experimental groups. Adult mice at least 10 weeks of age were used in all experiments. All experiments were approved by the Northwestern University Institutional Animal Care and Use Committee. An analysis of the influence of sex on our results was not provided given that our study is underpowered to detect potential sex differences.

### METHOD DETAILS

#### Stereotaxic surgery

Surgery was performed on adult (7–20 weeks old) male and female mice. Briefly, anesthesia was induced and maintained with isoflurane 1–4% (Patterson Scientific Link 7). Buprenorphine SR (0.5 mg/kg, Zoopharm) and Carprofen (5 mg/kg, Zoetis) were administered subcutaneously for analgesia. Ophthalmic ointment (Puralube, Dechra) was used to prevent dehydration of the cornea. A far infrared heating pad (Kent Scientific) was placed on top of the stereotax (Stoelting 51733D) to keep body temperature at ~37°C. Fur was removed with Nair; 10% povidone-iodine and 70% isopropyl alcohol were used to disinfect the scalp. A small (~1 cm) scalp incision was made to expose the skull, which was later closed with non-absorbable sutures (Ethicon, 661H) and tissue adhesive (Vetbond, 3M). Bregma and lambda were used as landmarks to level the head and guide injections. To drill skull holes, a micromotor drill (Stoelting, 51449) was moved to the appropriate coordinates with the aid of a digital stereotaxic display. Viruses and/or retrobeads were injected into the brain at 50-100 nl/min through a blunt 33-gauge needle using a syringe pump (World Precision Instruments). The needle was left in place for 5 min following the end of the injection, then slowly retracted to avoid leakage up the injection tract. The following coordinates were used (AP, ML, DV – in mm): DMS (0.8, 1.5, −2.8), DLS (0.3, 2.5, −3.3), and SNr (−3.3, 1.2, −4.7). Where indicated, we injected 250 nl of scAAV1-hSyn-Cre (2.81e13 vg/ml, WZ Biosciences) into DMS/DLS, and 250 nl of AAV5-hSyn-Con/Foff-EYFP (2.6e12 vg/ml, UNC, Addgene plasmid #55651) or AAV5-hSyn-Con/Foff-hChR2(H134R)-EYFP (5.3e12 vg/ml, UNC, Addgene plasmid #55646) into SNr. Red retrobeads (LumaFluor Inc) were diluted 1:4 (dilution factor) in sterile saline, and 100 nl were injected into DMS/DLS. When retrobeads were mixed with scAAV1-hSyn-Cre for investigation of closed loops, they were diluted 1:8 in a virus aliquot, and a total volume of 250 nl was injected into DMS or DLS. As a consequence, approximately the same amount of beads was injected into striatum (half the concentration at ~double the volume), and the transsynaptic Cre virus was only slightly diluted (7:8 dilution factor). After surgery, animals were placed on a warm recovery bin until ambulant. A moist nutritional supplement (DietGet 31M, Clear H_2_O) was placed on the floor of the homecage to aid recovery from surgery. 4-9 weeks after surgery, animals received a lethal intraperitoneal injection of Euthasol (1 mg/kg, Virbac), a combination of sodium pentobarbital (390 mg/ml) and sodium phenytoin (50 mg/ml), and underwent a transcardial perfusion for electrophysiology and/or histology experiments.

#### Electrophysiology

We followed the methods described by Ting and colleagues (Ting et al., 2014) to prepare acute brain slices from adult mice. Following Euthasol injection, unresponsive mice were transcardially perfused with ice-cold N-Methyl-D-Glucamine (NMDG) artificial cerebrospinal fluid (ACSF) containing (in mM): 92 NMDG, 2.5 KCl, 1.2 NaH2PO4, 30 NaHCO3, 20 HEPES, 25 Glucose, 5 Na-Ascorbate, 2 Thiourea, 3 Na-Pyruvate, 10 MgSO4, 0.5 CaCl2 (Millipore Sigma). All extracellular solutions used for electrophysiology were saturated with 95%O_2_/5%CO_2_ and their pH and osmolarity were adjusted to 7.3-7.4 and 300±5 mOsm, respectively. After perfusion, the brain was quickly removed and cut coronally to separate the rostral half (containing striatum) from the caudal half (containing SN). The cut face of each brain half was glued (Loctite 454) to a specimen holder and immersed into ice-cold NMDG ACSF. Coronal slices (300 mm thick) were made using a vibratome (Leica, VT1200S) set to 0.08 mm/s speed and 1.00 mm amplitude. Striatal slices were saved to confirm injection sites, while midbrain slices were used for recordings. Slices were allowed to recover for 45 min in three 15 min baths: (1) warm (33°C) NMDG ACSF; (2) warm (33°C) recovery ACSF, containing (in mM): 92 NaCl, 2.5 KCl, 1.2 NaH2PO4, 30 NaHCO3, 20 HEPES, 25 Glucose, 5 Na-Ascorbate, 2 Thiourea, 3 Na-Pyruvate, 1 MgSO4, 2 CaCl2; and (3) room temperature (RT) recovery ACSF. Finally, slices were kept at RT in recording ACSF, containing (in mM): 125 NaCl, 26 NaHCO3, 1.25 NaH2PO4, 2.5 KCl, 1 MgCl2, 2 CaCl2, 11 Glucose. During recordings, fresh ACSF was continuously delivered to the slice chamber at ~1.5 ml/min and warmed to 30-32°C with an inline heater (Warner Instruments). Where indicated, the following drugs were added to the recording ACSF: D-AP5 (50 μM, Cayman Chemical), NBQX disodium (5 μM, Tocris Bioscience), TTX (1 μM, Tocris Bioscience), 4-AP (100 μM, Tocris Bioscience), and GBZ (10 μM, Tocris Bioscience). Three different internal solutions were used in this study. For monosynaptic connectivity experiments, a high chloride internal solution was used, adjusted to 290±5 mOsm and pH 7.3-7.4, containing (in mM): 130 CsCl, 1 EGTA, 10 HEPES, 5 QX-314-Cl, 10 TEA-Cl, 2 Mg-ATP, 0.3 Na-GTP. For suppression of tonic firing experiments, a HEPES-buffered synthetic interstitial fluid solution (SIF) was used as internal solution, adjusted to 300±5 mOsm and pH 7.3-7.4, containing (in mM): 140 NaCl, 23 Glucose, 15 HEPES, 3 KCl, 1.5 MgCl2, 1.6 CaCl2. For pre-synaptic release probability experiments, a low chloride internal solution was used, adjusted to 290±5 mOsm and pH 7.3-7.4, containing (in mM): 130 CsMeSO_3_, 1 EGTA, 10 HEPES, 5 QX-314-Cl, 10 TEA-Cl, 2 Mg-ATP, 0.3 Na-GTP. Patch pipettes (3-5 MΩ) were pulled (Narishige, PC-100) from borosilicate glass (Warner Instruments, G150TF-4) and moved with the assistance of a micromanipulator (Sensapex). Cells were visualized with a 40x water-immersion objective (NA 0.8, Olympus, #N2667700) on a microscope (Olympus, BX51WI) equipped with infrared-differential interference imaging (DIC) and a camera (QImaging, Retiga Electro Monochrome). An LED light source (CoolLED, pE-300^white^) was used to illuminate the slice through the objective for targeted patching and for optogenetic stimulation. With the aid of a power meter (Thor Labs, PM130D), the LED power was adjusted to deliver ~10 mW/mm^2^ at 475 nm to the slice during the o-stim. Signals were recorded at 10 kHz using Wavesurfer v0.945 (https://wavesurfer.janelia.org/), a National Instruments Digitizer (NIDAQ X series PCIe-6323) and BNC Breakout (BNC-2090A), and a Multiclamp 700B amplifier (Molecular Devices). Data analysis was performed offline using custom-written MATLAB scripts.

#### Histology

The following protocol was used to slice, stain and image the tissue used exclusively for histology (Figures 2 and S2). Following Euthasol injection, unresponsive mice were transcardially perfused with ice-cold phosphate-buffered saline (PBS), followed by 4% paraformaldehyde (PFA) diluted in PBS. Brains were immersed in 4% PFA overnight, and then cryoprotected with 30% sucrose (diluted in PBS) at 4°C. Coronal slices (30-50 μm thick) were made using a freezing microtome (Leica, SM2010 R). Staining was performed on free floating slices, with 3x10 min PBS washes in-between incubations. Slices were blocked for 1-2 h at RT with 3% normal goat serum (NGS) diluted in 0.3% PBST (0.3% Triton X-100 in PBS). Then, slices were incubated overnight at 4°C with primary antibodies diluted in blocking solution. Striatum slices were incubated with guinea pig anti-Cre (1:500, Synaptic Systems, #257004) and rabbit anti-GFP (1:1000, Invitrogen, #A11122), while midbrain slices were incubated with chicken anti-TH (1:500, Aves Labs, #TYH) and rabbit anti-GFP (1:1000, Invitrogen, #A11122). Afterwards, slices were incubated for 2-3 h at RT in secondary antibodies diluted in a modified blocking solution (1% NGS in 0.3% PBST). Striatum slices were incubated with goat anti-guinea pig 647 (1:500, Invitrogen, #A21450) and goat anti-rabbit 594 (1:500, Invitrogen, #A11012), while midbrain slices were incubated with goat anti-chicken 647 (1:500, Invitrogen, #A21449) and donkey anti-rabbit 488 (1:500, Jackson Immuno Research, #711-546-152). Striatum slices were further stained for 1-2 h at RT with NeuroTrace 435/455 (1:100 diluted in PBS, Invitrogen, #N21479), a fluorescent Nissl staining. Fluoromount-G (Southern Biotech) was used as mounting media. Slides were imaged with an air-immersion 10x objective (NA 0.45, Nikon, #MRD70105) on an epifluorescence microscope (Keyence, BZ-X800).

A slightly different protocol was used to stain tissue derived from electrophysiology experiments (Figures 1, 3, 4, S1, S3, and S4). Slices were fixed overnight at 4°C in 4% PFA and stored in PBS at 4°C. Staining was performed on free floating slices as described above, with some modifications – 0.3% PBST was replaced by 0.5% PBST, 10% NGS was used for blocking, and 1% NGS was used to dilute antibodies. Cre staining was performed in striatum slices using guinea pig anti-Cre and goat anti-guinea pig 647. TH staining was performed in midbrain slices using chicken anti-TH and goat anti-chicken 647. EYFP signal was enhanced in all slices with GFP immunolabeling, using rabbit anti-GFP and donkey anti-rabbit 488. Retrobeads did not require enhancement. A custom look-up table was applied in ImageJ to match our colorblind safe color-coding (Wong, 2011). For qualitative visualization of midbrain slices, we adjusted the brightness and contrast of the retrobeads and EYFP channel separately due to the brighter fluorescence of the beads. Analysis of injection spread in DMS/DLS was performed in ImageJ (Schneider et al., 2012), using the following tools: threshold, median filter, and binary outline. A lower threshold was used for outlining the spread of retrobeads due to their brighter fluorescence in comparison to Cre immunolabeling, but the same analysis parameters were used for all mice. Images were aligned to two striatum sections from the Mouse Brain Atlas (Franklin and Paxinos, 2008), and injection outlines were superimposed in Adobe Illustrator.

### QUANTIFICATION AND STATISTICAL ANALYSIS

#### Monosynaptic connectivity

Bead-labeled cells were held at −70 mV and exposed to the o-stim (5 ms blue light pulse) in 5-10 sweeps, with a 30 s interval between sweeps. Series resistance (Rs) was monitored, but not compensated. Liquid junction potential was not corrected. Cells with Rs > 25 MΩ or with more than 30% change in Rs during the recording were excluded from the dataset. oIPSCs were characterized as fast-onset events (a monotonic decrease in current for 1.5 ms) that happened within 20 ms of the start of the light pulse. In rare sweeps, mIPSCs were mislabeled as oIPSCs. Thus, a *cell* was labeled as “shows an oIPSC” only if oIPSCs were detected in more than 50% of the recorded sweeps. Cells that did not fit this criteria were labeled as “no oIPSC”. For a subset of cells that showed an oIPSC, GBZ was added to the bath for 4 min, and the response to the o-stim was reassessed. Before testing a new cell, GBZ was washed off for at least 20 min. These wash-in and wash-off times were sufficient to block and unblock mIPSCs, respectively (data not shown). The oIPSC amplitude and onset latency reported for each cell were averaged across sweeps. In experiments using the VGAT-IRES-Cre line, a total of 17 cells (4 DLS-projecting and 13 DMS-projecting) were excluded from the dataset due to ChR2 expression, as evidenced by GBZ-insensitive oIPSCs with onset latency < 1 ms. A VGAT+ subgroup of dopaminergic neurons has been previously described (Poulin et al., 2020).

#### Suppression of tonic firing

Bead-labeled cells were recorded in voltage clamp (no holding voltage was applied) with a loose seal (20–100 MΩ) and exposed to the o-stim (5 ms pulses delivered at 20 Hz for 3 s) in 5–10 sweeps, with a 30 s interval between sweeps. 10 sweeps were recorded for 88% of the cells (126/142 cells). Cells that did not display tonic firing were excluded from the dataset. The baseline firing rate was calculated during the 3 s prior to the o-stim. Mean ± SD were calculated across sweeps. In our experiments using the VGAT-IRES-Cre line, a total of 16 cells (7 DLS-projecting and 9 DMS-projecting) were excluded from the dataset due to ChR2 expression, as evidenced by GBZ-insensitive light-evoked excitation.

#### Pre-synaptic release probability

Bead-labeled cells were held at −40 mV and exposed to the same o-stim used above (5 ms pulses delivered at 20 Hz for 3 s) in 5–10 sweeps, with a 30 s interval between sweeps. This holding voltage was chosen to allow detection of oIPSCs while masking spontaneous IPSCs. Series resistance (Rs) was monitored, but not compensated. Liquid junction potential was not corrected. Cells with Rs > 25 MΩ or with more than 30% change in Rs during the recording were excluded from the dataset. oIPSCs were characterized as fast-onset events (a monotonic increase in current for 0.5 ms) that happened within 5 ms of the start of the light pulse. For a subset of cells that showed oIPSCs, GBZ was added to the bath for 4 min, and the response to the o-stim was reassessed. Before testing a new cell, GBZ was washed off for at least 20 min. The oIPSC amplitude and onset latency reported for each cell (and light pulse) were averaged across sweeps.

#### Approximate cell location

Following each cell recording, a low magnification DIC image was taken with a 5× air-immersion objective (NA 0.15, Olympus, #N2181500) to show the relative position of the cell in the slice. Offline, images from the same slice were stitched in MATLAB for registration purposes. Stitched DIC images were later aligned to an MRI based atlas (Chon et al., 2019) in Adobe Illustrator and the relative coordinates of all cells was documented.

#### Statistical analyses

Most statistical analyses were performed in Prism (GraphPad) using the Kruskal-Wallis test, a non-parametric version of one-way ANOVA, followed by a Multiple Comparison Test. p < 0.05 was considered statistically significant. The p values were adjusted for multiple comparisons by controlling the False Discovery Rate (two-stage step-up method of Benjamini, Krieger and Yekutieli). A Multinomial logistic regression was performed in MATLAB to predict the likelihood of connection based on the medio-lateral, dorsal-ventral and antero-posterior location of the recorded cells. In all statistical tests, n represented the number of cells. The number of cells and mice used for each experiment is shown in the Figures.

## Supplementary Material

1

## Figures and Tables

**Table:** KEY RESOURCES TABLE

REAGENT or RESOURCE	SOURCE	IDENTIFIER
Antibodies		
Guinea Pig anti-Cre	Synaptic Systems	Cat# 257004; RRID:AB_2782969
Rabbit anti-GFP	Invitrogen	Cat# A11122; RRID:AB_221569
Chicken anti-TH	Aves Labs	Cat# TYH; RRID:AB_10013440
Goat anti-Guinea Pig 647	Invitrogen	Cat# A21450; RRID:AB_2735091
Goat anti-Rabbit 594	Invitrogen	Cat# A11012; RRID:AB_2534079
Donkey anti-Rabbit 488	Jackson ImmunoResearch Labs	Cat# 711-546-152; RRID:AB_2340619
Goat anti-Chicken 647	Life Technologies	Cat# A-21449; RRID:AB_2535866
Bacterial and virus strains		
scAAV1-hSyn-Cre	WZ Biosciences (Zingg et al., 2020)	Lot# 20200729
AAV5-hSyn-Con/Foff-EYFP	UNC Vector Core	Lot# AV6151; RRID:Addgene_55651
AAV5-hSyn-Con/Foff-hChR2(H134R)-EYFP	UNC Vector Core	Lot# AV8475; RRID:Addgene_55646
Chemicals, peptides, and recombinant proteins		
Red retrobeads IX	LumaFluor Inc	CAS: 78R180
D-AP5: D-APV	Cayman Chemical	CAS: 79055-68-8
NBQX disodium	Tocris Bioscience	CAS: 479347-86-9
TTX: Tetrodotoxin citrate	Tocris Bioscience	CAS: 18660-81-6
4-AP: 4-Aminopyridine	Tocris Bioscience	CAS: 504-24-5
GBZ: Gabazine: SR 95531 hydrobromide	Tocris Bioscience	CAS: 104104-50-9
QX-314-Cl: Lidocaine N-ethyl chloride	Sigma	CAS: 5369-03-9
TEA-Cl: Tetraethylammonium chloride	Sigma	CAS: 56-34-8
NeuroTrace 435/455	Invitrogen	Cat# N21479
Normal Goat Serum	Jackson ImmunoResearch Labs	RRID:AB_2336990
Fluoromont-G	Southern Biotech	Cat# 0100-01
Isoflurane	Henry Schein	CAS: 26675-46-7
Experimental models: Organisms/strains		
Mouse: VGAT-IRES-Cre: B6J.129S6(FVB)-Slc32a1^tm2(cre)Lowl^/MwarJ	The Jackson Laboratory	RRID:IMSR_JAX:028862
Mouse: TH-2A-Flpo: C57BL/6N-7*Th*^tm1Awar^/Mmmh	Awatranami Lab (Poulin et al., 2018)	RRID:MMRRC_050618-MU
Mouse: WT: C57BL/6J	The Jackson Laboratory	RRID:IMSR_JAX:000,664
Software and algorithms		
MATLAB R2020b	Mathworks	RRID:SCR_001622
Wavesurfer v0.945	HHMI Janelia	RRID:SCR_021529; https://wavesurfer.janelia.org
ImageJ, FIJI 1.53h	(Schneider et al., 2012)	RRID:SCR_003070; http://fiji.sc/
Prism 9	GraphPad	RRID:SCR_002798

## Highlights

- Transsynaptic and intersectional genetic tools to study striatonigrostriatal circuits
- Closed-loop striatonigrostriatal circuits can regulate dopamine neuron firing
- An open-loop “ascending spiral” circuit exists but does not regulate firing
- An open-loop “descending spiral” circuit also exists but does not regulate firing
