# Supplementary material for: Striatonigrostriatal circuit architecture for disinhibition of dopamine signaling
Source: Cell Rep. Author manuscript; Available in PMC 2022 Aug 30. (PMC9425427; doi:10.1016/j.celrep.2022.111228)
Supplement: 1 [file NIHMS1830645-supplement-1.pdf]

Cell Reports, Volume *40*

Supplemental information

Striatonigrostriatal circuit architecture for disinhibition of dopamine signaling

Priscilla Ambrosi and Talia N. Lerner

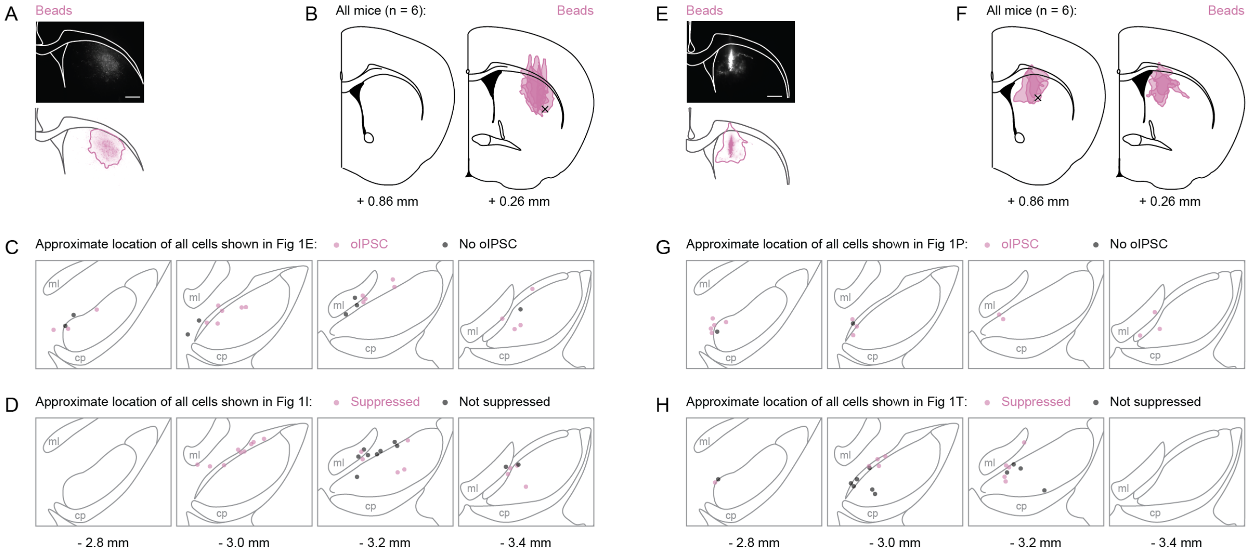

Figure S1. Injection spread in striatum and location of patched cells in midbrain slices for experiments with VGAT-IRES-Cre mice. Related to Figure 1. (A) Example striatum slice. Scale bar: 0.5 mm. **(B)** Approximate spread of retrobeads in the striatum of all mice used for Figure 1A-K. A black x marks the approximate target location for DLS injections. The numbers below the atlas images indicate their AP position relative to bregma. **(C)** Approximate location of all DLS-projecting cells recorded in whole-cell mode used for Figure 1E-H. Each dot is a cell, color-coded in magenta (oIPSC) or black (no oIPSC). **(D)** Approximate location of all DLS-projecting cells recorded in loose seal mode used for Figure 1I-K. Each dot is a cell, color-coded in magenta (inhibited) or black (not inhibited). The numbers below the atlas images indicate their AP position relative to bregma. **(E-H)** Same as A-D but relative to experiments shown in Figure 1L-V with DMS-projecting cells instead of DLS-projecting.

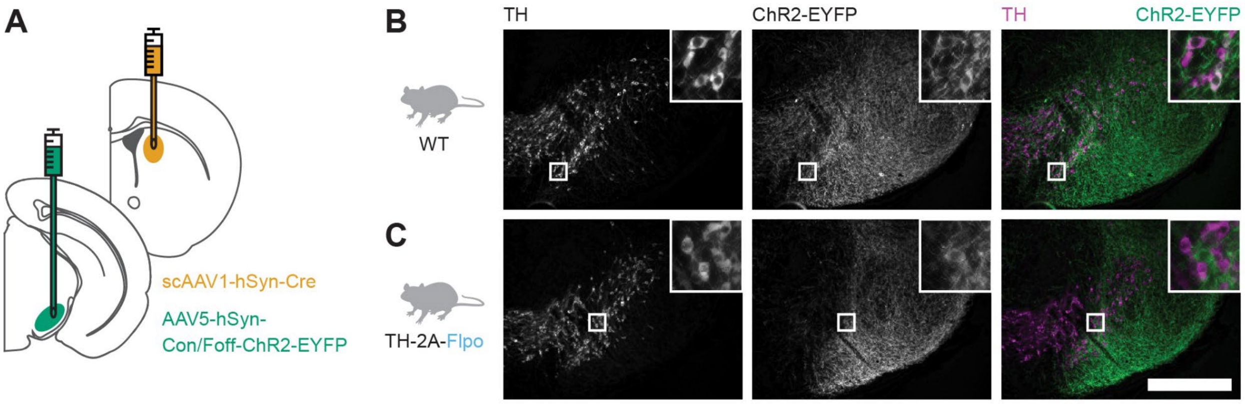

Figure S2. Similar results from Figure 2E are obtained using a Con/Foff-ChR2-EYFP virus. Related to Figure 2. (A) Experimental design for labeling DMS-targeted, non-dopaminergic neurons in SNr with ChR2-EYFP. **(B-C)** Example SN histology after injections in WT (B) and TH-2A-Flpo (C) mice. Scale bar: 0.5 mm.

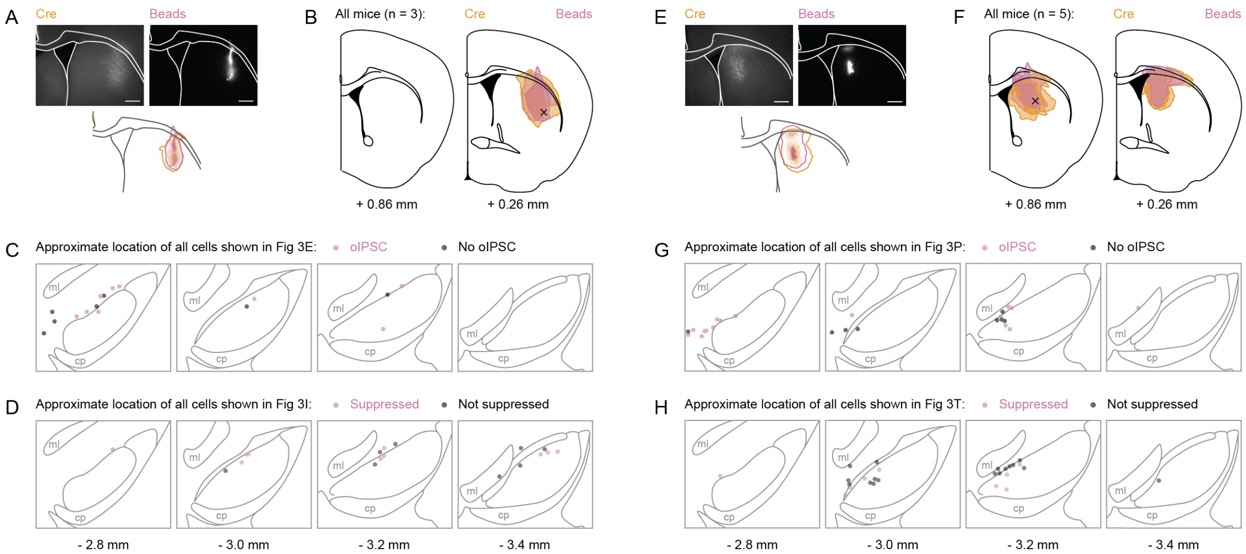

Figure S3. Injection spread in striatum and location of patched cells in midbrain slices for closed loop experiments. Related to Figure 3. (A) Example striatum slice. Scale bar: 0.5 mm. **(B)** Approximate spread of retrobeads in the striatum of all mice used for Figure 3A-K. A black x marks the approximate target location for DLS injections. The numbers below the atlas images indicate their AP position relative to bregma. **(C)** Approximate location of all DLS-projecting cells recorded in whole-cell mode used for Figure 3E-H. Each dot is a cell, color-coded in magenta (oIPSC) or black (no oIPSC). **(D)** Approximate location of all DLS-projecting cells recorded in loose seal mode used for Figure 3I-K. Each dot is a cell, color-coded in magenta (inhibited) or black (not inhibited). The numbers below the atlas images indicate their AP position relative to bregma. **(E-H)** Same as A-D but relative to experiments shown in Figure 3L-V with DMS-projecting cells instead of DLS-projecting.

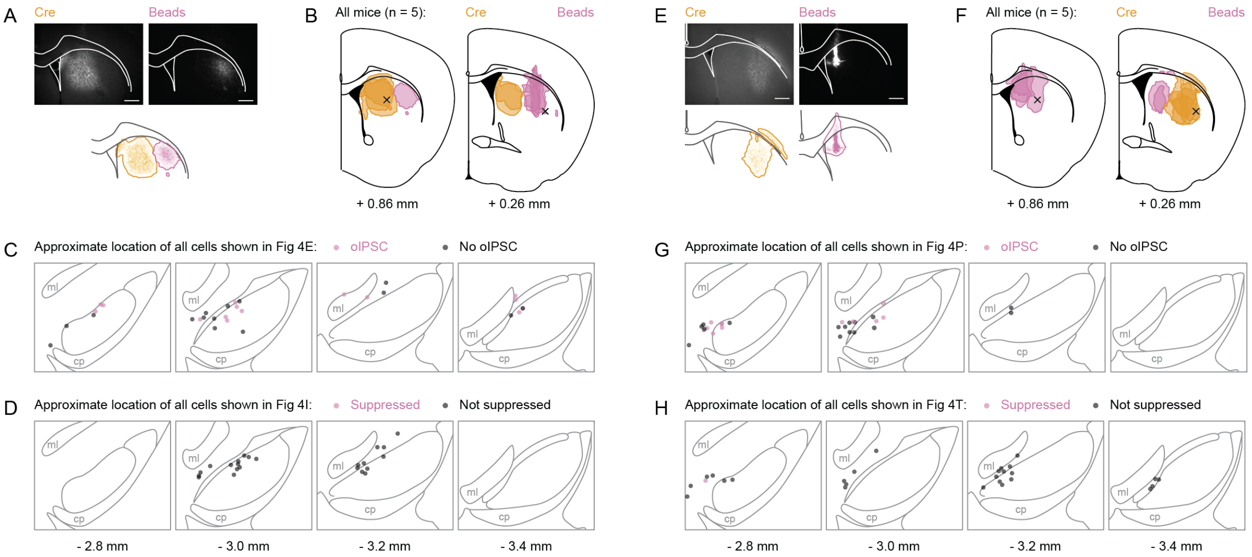

Figure S4. Injection spread in striatum and location of patched cells in midbrain slices for open spiral experiments. Related to Figure 4. (A) Example striatum slice. Scale bar: 0.5 mm. **(B)** Approximate spread of retrobeads in the striatum of all mice used for Figure 4A-K. A black x marks the approximate target location for DMS (left) and DLS (right) injections. The numbers below the atlas images indicate their AP position relative to bregma. **(C)** Approximate location of all DLS-projecting cells recorded in whole-cell mode used for Figure 4E-H. Each dot is a cell, color-coded in magenta (oIPSC) or black (no oIPSC). **(D)** Approximate location of all DLS-projecting cells recorded in loose seal mode used for Figure 4I-K. Each dot is a cell, color-coded in magenta (inhibited) or black (not inhibited). The numbers below the atlas images indicate their AP position relative to bregma. **(E-H)** Same as A-D but relative to experiments shown in Figure 4L-V with DMS-projecting cells instead of DLS-projecting.
